# Two Cases of Localised Necrosis of the Lung, with Recovery

**Published:** 1902-06

**Authors:** J. Michell Clarke

**Affiliations:** Professor of Pathology, University College, Bristol, and Physician to the Bristol General Hospital


### TWO CASES OF LOCALISED NECROSIS OF THE LUNG, WITH RECOVERY.

BY

J. Michell Clarke, M.A., M.D., F.R.C.P.,

*Professor of Pathology, University College, Bristol, and Physician to the Bristol General Hospital.*

I have called these cases localised necrosis of the lung, as the term seems to me to best embody the conditions present. Gangrene of the lung is by common consent applied to those cases in which the necrosis extends over a very wide area or involves all of one lobe of a lung. I am aware that many observers would class these cases as examples of localised bronchiectasis; and if we include under this term ulceration through the wall of the bronchus affected and destruction of an area of the neighbouring lung, this designation would be correct. But in calling such cases bronchiectasis, I think undue stress is laid on what is only a subsidiary and not the main part of the pathological process. Some irritant, generally of bacterial nature, lodges in a bronchus—as in the second of these two cases, where the pulmonary disease undoubtedly originated in the passage of a drop of pus from a peritonsillar abscess into a bronchus,—sets up inflammation, with ulceration, of the bronchial wall, and through this attacks the neighbouring lung tissue. In this there starts a patch of localised inflammation, which, if very intense, leads to necrosis of a small portion of lung or to the formation of an abscess in it. No doubt the affected bronchus undergoes dilatation either early from ulceration of its wall or later from contraction of the fibrous tissue which is formed in the attempt at repair of the mischief; but the chief cause of the symptoms lies in the changes in the surrounding lung tissue, and until these are removed no amelioration can result: hence I have preferred to lay stress on this part of the morbid process. It was on this view of the nature of the conditions that had to be dealt with that operation was resolved upon in each case; and it was thought that even if the exact seat of mischief were not reached, the inflammatory products might find their way through the intervening tissues into the drainage-tube, as had happened in other reported cases with succcessful results. This was also the fortunate result in my second case.

Probably the large amount of expectoration, of precisely the characters regarded as typical of bronchiectasis, depends on irritation by the highly toxic products of the diseased area of the mucous lining of the bronchi of the lower lobe of the lung, accompanied by acute dilatation of them. It would be difficult to account for such large quantities of sputum on any other view, and the sequel shows that this is the true explanation, and further that the condition is a temporary one; for when the conditions at the original source of disease have been relieved, these other (secondary) symptoms disappear. In both cases the absence of any of the usual causes which give rise to bronchiectasis and the acute character of the onset of the illness is noteworthy. In the first the cause of the lung mischief was obscure, and the relief must apparently be attributed to the intra-tracheal injections, which have been found effective in other such cases, as the improvement after exploration of the lung could hardly have been more than a coincidence.

H. C., æt. 47, an iron-plate worker, had always enjoyed good health, with the exception of an attack of rheumatic fever at the age of 20. It is important to note that he had never suffered from bronchitis, winter cough, or any chest affection. The present illness began five weeks previous to admission with pain in the back, which quickly passed to the right side of the chest, where it remained. He has suffered from cough, worse at night, and coming on in paroxysms, with the expectoration of a quantity of thick, viscid sputum, very offensive both to taste and smell. He was never sick with the cough, and occasionally the expectoration came up without any cough at all. He sweated profusely at night, and had lost flesh rapidly.

On admission he was pale, the appetite was bad, the tongue furred, the bowels loose, the temperature 102° F., the pulse 100, weak and compressible, the arteries somewhat thickened, and the respiration rate 24.

There was marked clubbing of the fingers. The cough was violent and paroxysmal, and the breath extremely offensive after a fit of coughing. He brought up a quantity of sputum, which was expectorated fairly easily, had the horribly offensive odour of the sputum in pulmonary gangrene, and on standing separated into four layers, the uppermost frothy, the next nummular greenish-yellow masses, in the third a watery fluid, and at the bottom a finer *débris*, which consisted, under the microscope, of small portions of lung-tissue, of large squamous cells, smaller epithelial cells and pus-cells. Most of these cells had undergone marked fatty degeneration; there were no crystals of the fatty acids, and tubercle bacilli were absent.

On examination of the chest there was harsh breathing over the front of the right lung, and at the back there was an area of dulness extending from the fifth rib above to the ninth rib below, outwards as far as the posterior axillary line, inwards to the spine. Over the upper part of this area, as low as the lower angle of scapula, the breath-sounds were harsh to bronchial, with spongy or sticky crepitant râles, and the vocal resonance increased. Below this both breath-sounds and vocal resonance were diminished. In the sixth space, one inch from the spine, was an area the size of a crown, over which was absolute dulness, cavernous breath-sounds, bronchophony, and whispering pectoriloquy. Over the whole of the above area of dulness the chest-wall was œdematous. The left lung was normal.

The heart and abdominal organs were healthy. The urine, of low specific gravity, contained no albumin or sugar. There was some kyphosis of the upper dorsal spine.

He was ordered a nourishing diet with port wine, a stimulant expectorant mixture, and intra-tracheal injections twice daily of creosote, menthol, and olive oil.

During the next ten days the cough was very bad, disturbing his sleep; the expectoration, which was abundant, and the breath, were horribly offensive; the pulse was weak, varying from 100—120; the respirations were 28; and the temperature very irregular, ranging from 99° in the morning to 102° or 103° at night. The characters of the sputum remained as given above, and the physical signs showed no alteration. At the end of this time, as the patient was steadily losing ground as regards his general condition, and as the physical signs and symptoms pointed to a patch of necrosis of the lung or a small abscess within it, it was decided to make an attempt to reach and drain the seat of mischief.

Under an anæsthetic Mr. Morton therefore carefully inserted the large needle of an exploring syringe in several places over the dull area at the back of the right lung; but, failing to find pus in any one of them, decided to do no more at that time. During the evening the patient coughed up a quantity of blood-stained offensive sputum, but in other respects was no worse. During the following two days the temperature gradually fell to normal, and the patient felt easier and was free from pain. The intra-tracheal injections were continued, and in addition creosote ♏ v. was given every four hours by the mouth.

On August 10th (five days after exploration) the temperature was normal, after rising to 101.5° on each of the two previous nights. The fœtor of the sputum was less marked, the conditions as regards sputum and condition of lung much the same.

On August 17th the general condition of the patient was much improved: the temperature had remained normal, the tongue was cleaner, the pulse improved, the cough less, only occurring now in paroxysms at long intervals, the expectoration less abundant, less fœtid, but still separating into layers containing the same elements. A friction sound was now audible over the lower part of the area of dulness.

From this time uninterrupted improvement took place both in the general and local conditions. On Sept. 3rd a note was made that the patient had been up in the ward for four days, the cough was much diminished, and the violent paroxysms had not occurred for a week. The sputum had greatly lessened in amount, and the fœtor had entirely gone. The physical signs also showed marked improvement; there was still a faint rub, the area of dulness had given place to a much smaller area of deficient resonance along the posterior border of the scapula; the breath-sounds were harsh, but nowhere bronchial; all crepitus had disappeared, and there was only an occasional rhonchus. He was rapidly regaining strength and putting on flesh.

On Sept. 10th still further improvement was noted, and shortly afterwards the patient left the Hospital practically well, with the cough almost gone and expectoration entirely so, and on physical examination only a small patch of deficient resonance and breath-sounds internal to the posterior border of the scapula. It should be added that the intra-tracheal injections were continued during the stay in Hospital after the operation.

H. K., æt. 38, was first seen in February, 1901, with the late Dr. Wilding. He was then suffering from severe cough, coming on in paroxysms, and frequently ending in vomiting, with the expectoration of horribly offensive sputum, amounting in quantity to a pint or 1½ pints in the twenty-four hours. The history of the illness was that until June, 1900, he had always enjoyed good health; but he then became very ill with a bad sore throat, resulting in a peritonsillar abscess on the right side. This was opened, and during evacuation of the abscess some of the discharge, which was exceedingly foul, passed into the trachea, and he felt it trickle down into the right lung. The abscess healed, but three weeks afterwards he began to suffer from violent attacks of cough, with expectoration of offensive matter, often ending in vomiting. If the cough came at meal-times the food was vomited. This first attack lasted three months, and he then had a fortnight's remission, to be followed by a second illness lasting two months. After that he remained more or less free from cough, and was able to resume his work as a clerk for nearly four months. The present illness has lasted three weeks.

In each case the cough came in violent fits lasting 10—15 minutes, frequently ending in vomiting; between these violent paroxysms there was a continual slighter cough, with fœtid expectoration. During the week previous to my visit he had had one paroxysm of cough every twenty-four hours, generally at night, when he coughed up about a pint of horribly offensive sputum. I saw this. It separated into the typical three layers, and had rather the indescribably offensive odour met with in gangrene of the lung than the stale, fœtid odour of bronchiectasis.

The patient had lost nearly a stone in weight, he looked sallow and cachectic, sweated profusely at night, the temperature was occasionally raised in the evening; there were no shivering fits. As under his present conditions he was obviously going downhill, and there could only be one termination to his illness unless some more effective measures could be taken to arrest the disease, I advised him to come into the Hospital.

On admission the chest was hyper-resonant in front, moved fairly, measured 30″ expn. to 32½″ insp. The breath-sounds were harsh, and attended with slight rhonchus at inspiration, especially at the right apex. Vocal resonance, a trifle increased. Over the back the signs were the same, except that on the right side there was a patch of dulness beginning above at seventh rib and extending to tenth rib below; transversely it began 3″ from the dorsal spines, and extended 3″ outwards. It was roughly quadrilateral in shape. Over the upper part of this area the breath-sounds were exaggerated with prolonged expiration; occasionally indistinct soft crepitus and increased vocal resonance. The other thoracic and abdominal organs were normal; the urine contained a trace of albumin.

He was put on a nourishing diet, given creosote in increasing doses, and intra-tracheal injections of creosote, menthol, and olive oil twice daily. With the exception of slight diarrhœa for the first three weeks of his stay in Hospital, he improved remarkably, the cough was very slight, and the sputum, though slightly offensive, was insignificant in quantity. He did not, however, attach much importance to this improvement himself, as he told us that he had had the same experience before, and then, without apparent cause, had relapsed to be as bad as ever.

On March 28th the cough became much worse, the paroxysmal attacks returned, though not so badly as before, and he brought up again a quantity of very offensive, brownish sputum, separating into three layers, and containing red blood-cells, pus-cells, squamous epithelium, fat droplets, but no elastic tissue or tubercle bacilli. There were some long bacilli of uncertain nature. His temperature rose to 100° F. at night, he lost his appetite, felt ill and weak, could not sleep for cough, and for the same reason could not lie down, but was obliged to sit propped up with pillows. He had pains under the right shoulder-blade, and the physical signs at the right base were more definite, the dulness being more decided and the breath-sounds more bronchial in character. There was leucocytosis, the white cells numbering 27,000 to c.m.

Throughout the illness he stated that he felt the intra-tracheal injections trickle down into the right lung to about the same spot where he had felt the pus when the peri-tonsillar abscess was opened, and each injection appeared to reach the same spot.

My view of the case was that some of the pus from the peri-tonsillar abscess had passed down into the lower lobe of the right lung, and had, probably from its containing virulent micro-organisms, caused necrosis of a portion of the lung, possibly going on to formation of an abscess at the termination of the bronchus in which it had lodged, and to this necrosis or abscess the symptoms were due. Experience of other cases further led me to believe that the pus had passed down that long branch of the main bronchus to the lower lobe that runs in a direction almost directly downwards and slightly backwards for a long distance in the posterior border of the lung, not far from its posterior surface, to terminate almost at the lower border. I therefore asked Mr. Lansdown to see the case with a view to operation. We previously on the cadaver determined the relation of the lower part of this bronchus to the chest-wall, and also found that a needle passed vertically through a spot on the chest-wall corresponding to about the middle of the dull area above described would lead directly to this bronchus; this latter point tended to confirm the view that I had formed as to the position of the disease.

On April 14th Mr. Lansdown therefore resected a portion of the ninth rib in the line of the posterior border of the scapula (arm lying by side). The lung was not felt to move beneath the pleura in this position; but as there were no adhesions, nothing more was done at this time than to stitch the parietal and visceral layers of the pleura together. The next day the patient brought up a quantity of thick, blood-stained, foul-smelling sputum, and during the ensuing week he suffered a good deal from cough and brought up a little sputum. The temperature on three nights rose to 100°. By the end of the week he was better; the wound had been packed with green protective, and remained quite healthy.

On April 23rd a needle was passed in several directions into the lung, but no pus was found; an incision was made into the lung for a depth of one inch, and a drainage-tube inserted. During the next two days he coughed up a good deal of blood-stained offensive expectoration.

On April 27th the dressing was soaked with pus, which had the same offensive smell as the sputum. From this time there was a constant discharge of pus from the lung, which steadily decreased in quantity until, by May 20th, it was very small in amount. Cultures made from the pus showed the presence of staphylococcus aureus and of a bacillus which almost exactly resembled the diphtheria bacillus in morphology and staining properties. The patient now rapidly improved in health and strength, put on flesh, had very little cough, lost his pain in the right side, and the sputum ceased to be offensive and was very small in amount. On May 29th he went to the Convalescent Home. On June 13th he was readmitted into the Hospital, as he was not getting on well. There was very little pus discharged through the drainage-tube, but he was again coughing up a large quantity of frothy, greenish-yellow, blood-stained sputum. The cough was troublesome; he again began to lose appetite and flesh. Temperature normal. Physical signs in chest much the same. No albumin in urine.

On June 19th Mr. Lansdown reopened the wound, and explored the cavity with his finger. No pus was evacuated, but it was found that the cavity communicated freely with a bronchus. A very large drainage-tube was inserted. The temperature remained normal, and the bad odour and taste disappeared from the sputum, which again diminished in amount, whilst there was free discharge of pus from the wound.

By July 1st the discharge from the wound was much less, he was able to get about and to go outdoors. The cough varied, some days being troublesome, on others less so. He had a good deal of pain in the back.

On July 3rd cultures of the pus from the wound showed the presence of a bacillus exactly resembling the Klebs-Loeffler bacillus, in addition to staphylococcus aureus. Cultures taken on the two following days confirmed presence of this bacillus. It was noticed on dressing the wound that a soft thin fibrinous membrane could be withdrawn from the sinus, and on this account he was given four injections on successive days, each of 4,000 units antitoxin.

It should be stated that he had had no sore throat since his admission into the Hospital. The bacilli, which so closely resembled diphtheria bacilli, were found in pure culture in the thin fibrinous membrane on the edges of the wound; none were found in the sputum. The membranous material was not so tough and thick as the ordinary membrane seen in diphtheria. During all this month (July) he was very ill. His temperature remained normal, the pulse varied from 90—100 and was weak, he lost flesh and strength, the cough was troublesome. On the other hand, the sputum was very small in quantity, and was not offensive.

Towards the end of the month the discharge from the wound had become very slight or had practically ceased; but he was obviously losing in strength, and suffered much from the heat of the weather and from loss of appetite. He was very anxious to go home, and as the wound had by July 26th practically healed, I allowed him to do so, though with considerable misgivings as to his future; for in spite of the improvement with regard to cough, nature and quantity of sputum, he looked as if he would not recover. I had, however, some hope that he might derive benefit from change of air.

I did not see him again, as I went away for my holiday; but he came up to see me at the end of October, and had then quite recovered. He told me the wound healed speedily after leaving the Hospital, he lost all cough and expectoration in 4—6 weeks' time, and had since remained free from them; he looked much stronger, and said he was in very good health. In the chest the only signs were a little deficient movement, resonance, and breath-sounds over the right base.

There are one or two points of interest in this case. One is, Had he true diphtheria, and did he contract diphtheria of the wound? It is *a priori* difficult to see how he could have done so, as the wound was dressed antiseptically throughout and never left uncovered. Only on one or two occasions was there sufficient discharge of pus to soak through the dressings, and then they were immediately changed. Further, there has not been a case of diphtheria in the ward in which he lay for some years.

In gangrene of the lungs an organism is found which, morphologically, exactly resembles the diphtheria bacillus. Babes[1] mentions this organism as amongst those most frequently found in cases of pulmonary gangrene, and speaks of it as a pseudo-diphtheria bacillus. The absence of neuritis, of any affection of the kidneys or heart, is against infection by the true diphtheria organism, as if such secondary infection had occurred the probability would be that the patient, in his already enfeebled state, would have been especially prone to suffer in one or more of these ways.

However, in view of the presence of this bacillus and the formation of membrane, I thought it advisable to administer a few injections of antitoxin. I may say in passing that the membranes were thin and soft, never tough or fibrous. They were moderately adherent to the raw surface.

Is it possible that the lung was infected from the throat, and that with the increased formation of pus after an opening was made into it the bacillus had an opportunity to grow and develop?

Another point is the indefinite character of the local signs in the lungs, probably in part due to the diseased area never reaching to the surface. The constitutional symptoms were therefore of great importance, and I considered when I saw him first that unless by some means the diseased spot could be more directly attacked the disease would progress more or less quickly to a fatal termination.

[1] *Journ. of Path. u. Bacteriol.*, Bucharest, vol. vi.

